# Experimentally Validated Model Accounts for T Cells' Discriminating Ways

**DOI:** 10.1371/journal.pbio.0030391

**Published:** 2005-10-25

## Abstract

.

When a pathogen slips into the body, it might infect a cell or get eaten by a specialized white blood cell. Either way, its proteins get chopped into peptide fragments, loaded onto molecules encoded by genes in the major histocompatibility complex (MHC), and sent to the cell surface as a peptide-loaded MHC molecule (pMHC) for immune surveillance. The immune system can rally against billions of pathogens, in part because every T cell expresses a unique receptor (TCR), acquired during development, that recognizes specific pMHCs. Developing T cells undergo a selection process that weeds out more than 98% of cells, leaving only those cells whose receptors react to “self” pMHCs enough to signal but not enough to fully activate the T cells. Because antigen-presenting cells (APCs) in an infected host bear both self and pathogen-derived pMHCs, proper immune function depends on the discriminatory capacity of TCRs: they must allow a full T cell response to foreign antigens and avoid reacting to self-peptides on the same cell surface.

When a T cell homes in on a pathogenic antigen, the response is quick, sensitive (just a few molecules can set them off), and digital (all or nothing), engaging signaling pathways necessary for T cell activation, proliferation, and survival. (Molecules called agonist ligands trigger TCR signaling.) Signals relay through the cell as kinase enzymes activate proteins by triggering chemical reactions that add one or more phosphates (a process called phosphorylation); removing phosphates (called dephosphorylation) deactivates the proteins and the signal decays.

One explanation for this rapid, sensitive response is that pMHCs induce unique conformational changes in the receptor to initiate signaling. But a TCR can react differently to the same pMHCs at distinct points in its life history, and binding pairs show no conformations specific to agonists versus non-agonists. Alternately, in the kinetic proofreading concept, a kinetic threshold related to the TCR–pMHC binding properties accounts for ligand discrimination—though in simple forms of this hypothesis discrimination occurs at the expense of a rapid, sensitive, digital response.

Hoping to resolve these discrepancies, Grégoire Altan-Bonnet and Ronald Germain combined computer modeling with experimental results to develop a quantitative model of early TCR signaling. The authors revealed a novel aspect of TCR signaling—the explosive digital response of a key enzyme in the pathway—and identified competing feedback systems that may explain how T cells combine selectivity with a rapid, sensitive response.

To construct a predictive model, Altan-Bonnet and Germain measured kinetic aspects of biochemical responses to TCR–pMHC pairing by focusing on extracellular signal-related kinase (ERK), a key player in the TCR signaling cascade. Using T cells harvested from transgenic mice engineered to produce identical TCRs and APCs engineered to express very few self-pMHCs, the authors studied the conditions required for ERK activation. In individual T cells, the ERK pathway was activated by as few as ten foreign ligands. These experiments also revealed a “previously unappreciated aspect” of ERK signaling in T cells: it either occurred or it didn't, but when it did, the result was 100,000 phosphorylated ERK enzymes.

T cells that showed this explosive response could also distinguish between pMHCs with minor differences in TCR binding time, reinforcing the importance of the SHP-1 negative feedback system in preventing “sneak-through activation” by non-agonists. (SHP-1 is a dephosphorylating enzyme.) To test this hypothesis in a quantitative manner, Altan-Bonnet and Germain constructed a TCR signaling model in which kinetic proofreading of TCR–ligand interactions produces a quick initial negative feedback response (mediated by SHP-1) and a delayed, explosive digital ERK positive feedback system that, once activated, overrides the negative pathway and allows productive signaling. The authors then ran signaling simulations using different ligand numbers with different TCR binding lifetimes. Their model “shows almost absolute discrimination” between closely related pMHCs while preserving a fast, sensitive response to just a few agonist ligands. [Fig pbio-0030391-g001]

**Figure pbio-0030391-g001:**
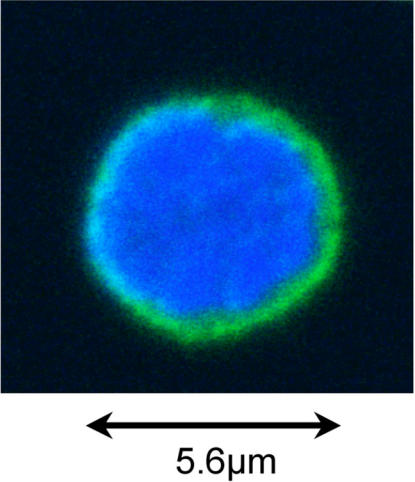
Confocal microscopy was used to determine the cytoplasmic volume of T cells to infer concentrations of signaling molecules in the cytoplasm

The model also yielded predictions that the authors validated experimentally: the ERK response slows down dramatically at low ligand densities; negative feedback adjusts to ligand strength and quantity to prevent signaling by high concentrations of low-affinity ligands, and allows sensitive responses to low concentrations of high-affinity ligands; differential activation of the negative feedback explains the existence and hierarchy of antagonism in T cell activation; and mature differentiating T cells permit signaling with different levels of ligand discrimination, depending on intracellular concentrations of molecules such as SHP-1.

Altogether, these results suggest that ligand discrimination is not “hard-wired” into TCR–ligand structural affinities. Rather, the threshold that permits TCR signaling varies as concentrations and dynamics of intracellular molecules vary during T cell development and after antigen activation. The model described here, though a necessarily simplified version of TCR signaling, highlights the effectiveness of simple feedback loops in helping cells filter out unwanted signals while effecting quick, sensitive responses—properties crucial for most other regulatory networks. The model also reinforces the importance of detailed probing of cell signaling dynamics to better understand the functions of a living system. —*Liza Gross*

